# Retear of anterior cruciate ligament grafts in female basketball players: a case series

**DOI:** 10.1186/1758-2555-2-7

**Published:** 2010-03-09

**Authors:** Yoshinari Tanaka, Yasukazu Yonetani, Yoshiki Shiozaki, Takuya Kitaguchi, Nozomi Sato, Shinya Takeshita, Shuji Horibe

**Affiliations:** 1 Department of Orthopaedic Surgery, Osaka Rosai Hospital, Sakai, Japan; 2 Department of Rehabilitation, Osaka Rosai Hospital, Sakai, Japan; 3 Department of Orthopaedic Surgery, Seifu Hospital, Sakai, Japan

## Abstract

**Background:**

Incidence of anterior cruciate ligament (ACL) injuries in young female basketball players is higher than that in male basketball players. Graft retears are more frequent with the increasing number of ACL reconstructions. The present study aimed to examine the incidence of retears in competitive female basketball players.

**Methods:**

Sixty-four female basketball players (aged 12 to 29 years) who underwent primary anatomic double-bundle ACL reconstruction using hamstring grafts participated in the study. We investigated incidence, mechanism, and patient characteristics of ACL graft retears. Mann-Whitney *U *test was used for statistical analysis, and the level of significance was determined at *P *< 0.05.

**Results:**

Six patients suffered from ACL graft retear (9.4%). Mean duration between primary ACL reconstruction and incidence of retears was 11.7 months. However, there were no other postoperative graft ruptures after 24 months. Primary injury and retear mechanisms varied by patient. At six months after the primary ACL reconstruction surgery, mean quadriceps and hamstring strengths were 81% and 87%, respectively, indicating favorable recovery of muscle strength. However, preoperative quadriceps and hamstring strength in the retear group were 65% and 71%, respectively. In particular, preoperative quadriceps strength in the retear group demonstrated a lower value than that in the uninjured group (*P *< 0.05).

**Conclusions:**

We observed a high incidence of ACL graft retears in competitive female basketball players, as previously reported. Considering the timing of graft retear occurrences, an early return to playing basketball should be avoided following ACL reconstruction. Closer attention should be paid to player preoperative condition, as well as muscle strength and postoperative status.

## Introduction

Anterior cruciate ligament (ACL) rupture is a disabling knee injury which frequently occurs in young athletes. Previous studies have critically assessed risk factors for primary ACL injury, including variables such as gender, levels of sports activity, and anatomical characteristics [1,2]. Female to male ratio of ACL injuries in basketball players was 3.6 and 4.5 in high school and in college, respectively [3].

ACL reconstruction is currently the gold standard to restore knee function after ACL rupture [4], but long-term efficacy has not been fully established [5]. ACL reconstruction using hamstring tendons has become a popular procedure because of its lower risk of donor site morbidity [6-8]. In addition, recent improvement in operative procedures has made it possible to perform anatomical double-bundle ACL reconstruction. This offers several advantages over the traditional Rosenberg's one or two femoral sockets ("bi-socket") procedure, including better biomechanical outcomes and more favorable clinical results [9-11].

However, with the increasing number of ACL reconstructions, graft failures have become more frequent. Rate of graft failures is reported as high as 8% of primary ACL reconstructions [12], and causes for failures can be classified into three categories: technical errors, biological failures, and traumatic failures. However, risk factors of graft retears remain unknown [13,14], and a single study has examined the risk factors for ACL graft retears [15]. The authors described the return to competitive sports as requiring movements such as side-stepping, pivoting, and jumping, and playing basketball was one risk factor for repeated ACL injury. Despite these facts, most athletes hope to return to sports activity following ACL reconstruction. The present study aimed to examine the incidence of ACL graft retear in female basketball players.

## Methods

### Patients

Between January 2004 and December 2006, primary anatomic double-bundle ACL reconstruction was performed on 104 knees in 101 female basketball players. Of those, 64 knees in 64 patients were included in the current study according to the following inclusion criteria:

1. Patient had a normal contralateral knee at the primary ACL reconstruction.

2. Patient underwent ACL reconstruction within 18 months after the injury.

3. Preoperative and postoperative muscle strength data were collected with the use of a Cybex II dynamometer (Lumex, Ronkonkoma, NY).

4. Patient received postoperative follow-up for at least eight months, at which point they were permitted to return to their sports activity.

5. Patient regained knee stability at six months after the surgery. Mean age of the patients was 16.2 years (range: 12 to 29 years) at the time of primary ACL reconstruction. Preoperatively, 59 patients (92.2%) played basketball at a competitive level (Table 1). Postoperatively, 35 patients (54.7%) returned to play at a competitive level and 13 patients (20.3%) played recreationally, while 11 patients (17.2%) did not return to play because of school graduation (Table 1).

**Table 1:** Preoperative and postoperative activity levels

Activity levels	Preoperative (n)	Postoperative (n)
Competitive	59	35
Vigorous recreational	3	7
Light recreational	2	6
ADL	0	11
Unknown	0	5

### Surgical Procedure

We performed anatomic double-bundle ACL reconstruction using hamstring autografts [16]. Autogenous semitendinosus tendon grafts were used for graft materials. After identification of the femoral and tibial footprints of the ACL, two 2.4 mm guide pins were inserted from the lateral femoral cortex to points between the resident's ridge and the posterior margin of the articular cartilage using the anterolateral entry femoral aimer (Smith & Nephew Endoscopy, MA). For the tibia, a 2.4 mm wire was inserted into the center of the posterolateral fiber at an angle of approximately 55° to the sagittal plane and 10° to the tibial axis using the guide, and another 2.4 mm wire was inserted into the center of the anteromedial fiber at an angle of approximately 45° to the sagittal plane and 20° to the tibial axis also using the guide. Each wire was overdrilled with a drill bit of appropriate (5-6 mm) diameter. Posterolateral and anteromedial grafts were fixed with EndoButton (Smith & Nephew Endoscopy, MA) to the femur. Two double-spike plates (DSP; MEIRA Corp., Nagoya, Japan; US Patent No. 6117,139,21) were used for the tibial fixation [17]. An initial tension of 1 MPa (approximately 20 to 25 N for each graft) was applied. After retightening the tension suture by repetitive manual pulling to remove stress relaxation, each graft was fixed at 15° to 20° of knee flexion with DSP and cancellous screws.

### Postoperative Rehabilitation and Return to Basketball

Patient knees were immobilized with braces postoperatively for two weeks. We allowed partial weight bearing at three weeks, and full weight bearing at four weeks. Jogging and running were allowed at three and four months, respectively. Patients were allowed to return to their previous activity levels after eight to ten months if postoperative quadriceps and hamstring strength levels of their injured leg improved to approximately 85% and 80%, respectively, at six months. None of the patients complained of subjective instability after their return to play. In addition, we observed no evident objective instability as assessed by the Lachman test, pivot shift test and KT-2000 arthrometer. Side-to-side difference of the anterior laxity at maximum load measured by KT-2000 was less than 2 mm in all cases.

### ACL Graft Retear

Graft retear was defined as follows:

1. Patient experienced an evident traumatic episode on the operated knee.

2. The knee became unstable after the re-injury.

3. Magnetic resonance imaging (MRI) confirmed the graft rupture.

### Muscle Strength Testing

We assessed the strength of the quadriceps and hamstring at 60° per second using a Cybex II dynamometer, both preoperatively and six months after surgery. Peak torque value was calculated and strength was expressed as a percentage of the uninvolved limb. Body weight ratio (BWR) for each muscle strength as well as the hamstring to quadriceps (HQ) ratio were also recorded.

### Statistical analysis

Statistical analysis was performed using SPSS for Windows software (SPSS Inc., Chicago, IL). Mann-Whitney *U *test was used to examine the differences between the uninjured and retear groups. The level of significance was determined at *P *< 0 .05.

## Results

### Incidence of retear of the ACL grafts in competitive female basketball players

**Figure 1:**
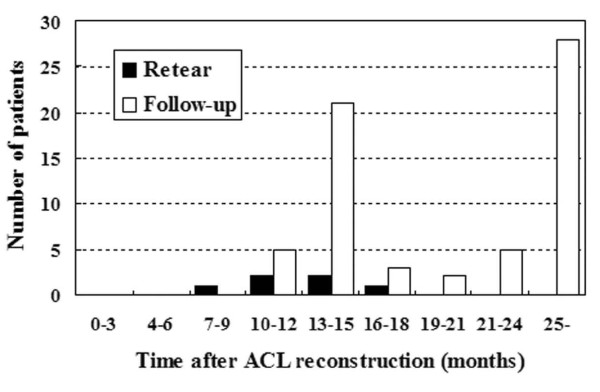
**Number of patients with ACL graft retear (black bars) and those who received follow-up (white bars) after reconstruction**.

Of the 64 patients, ACL graft retears occurred in six patients (9.4%). Mean duration between the index operation and the retears was 11.7 months (range: 8.0 to 15.7 months). Follow-up with 28 patients (43.8%) over a 24-month period revealed that none of these experienced graft ruptures (Figure 1).

### Mechanism of graft retears

The most common maneuvers that caused the primary injuries were landing (36.0%), stopping (18.8%), and cutting (18.8%), comparable with those described in previous reports [18]. Mechanisms for primary injury and graft retear are described in Table 2. None of the patients sustained re-injury by the same mechanism that caused the primary injury. Moreover, we observed an increased number of retears caused by the cutting maneuver mechanism.

**Table 2:** Mechanisms of primary injury and retear

Patient	Primary injury	Retear
1	Landing	Cutting
2	Direct blow	Cutting
3	Stop	Direct blow
4	Landing	Direct blow
5	Unknown	Cutting
6	Stop	Landing

### Characteristics of patients with retears

Patient demographics in the retear group are summarized in Table 3. Mean preoperative period for retear patients (1.9 months, range: 1.5 to 2.9 months) was shorter than that for uninjured patients (4.4 months, range: 0.7 to15.1 months), but this difference was not statistically significant (*P *= 0.11).

**Table 3:** Patient demographics

	Total patients	Injured patients
Total number of patients	64	6
Total reconstructions performed	64	6
Age at the time of surgery (year) (range)	16.2 (12-29)	15.8 (14-18)
BMI (range)	21.7 (17.6-27.2)	20.8 (18.9-23.9)
Preoperative period (months)	4.2	1.9
Time between operation and returning to sports	9.9	9.8
Meniscal lesion		
Medial meniscus	23 (35.9%)	1 (16.7%)
Lateral meniscus	26 (45.3%)	3 (50.0%)
Cartilage lesion	19 (29.7%)	1 (16.7%)

**Figure 2:**
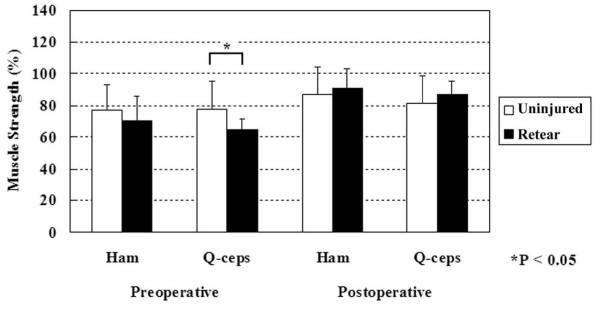
**Hamstring (Ham) and quadriceps (Q-ceps) preoperative and postoperative strength**.

Cybex II dynamometer measurements are reported in Table 4. Mean quadriceps and hamstring strength at six months after surgery were 81% and 87%, respectively, indicating favorable recovery of muscle strength. Preoperative quadriceps and hamstring strength of the injured leg in the retear group were lower than those in the uninjured group (Figure 2). In particular, preoperative quadriceps strengths in the uninjured and retear group were 78% and 65%, respectively, with a statistically significant difference (*P *< 0.05). Preoperative hamstring strength values in the uninjured and retear group were 77% and 71%, respectively, with no statistically significant difference (*P *= 0.13) (Figure 2).

**Table 4:** Preoperative and postoperative Cybex II testing data

	Preop.	Postop.	*P*
Ham. peak torque (Nm) (uninvolved)	58.5 ± 10.6	64.8 ± 14.0	0.01
Ham. peak torque (Nm) (involved)	44.7 ± 11.6	56.6 ± 16.6	< 0.01
Ham. BWR (%) (uninvolved)	105.9 ± 18.9	117.3 ± 24.5	< 0.01
Ham. BWR (%) (involved)	81.0 ± 21.3	102.5 ± 29.2	< 0.01
Q-ceps. peak torque (Nm) (uninvolved)	121.2 ± 23.6	134.7 ± 27.2	< 0.01
Q-ceps. peak torque (Nm) (involved)	91.3 ± 21.7	109.7 ± 29.3	< 0.01
Q-ceps. BWR (%) (uninvolved)	219.2 ± 40.7	244.1 ± 48.6	< 0.01
Q-ceps. BWR (%) (involved)	165.3 ± 38.2	198.6 ± 51.4	< 0.01
Ham. strength (%)	76.7 ± 16.0	87.3 ± 17.0	< 0.01
Q-ceps. strength (%)	76.4 ± 17.2	81.7 ± 16.6	0.01
H/Q ratio (%) (uninvolved)	49.1 ± 7.7	48.6 ± 7.8	0.63
H/Q ratio (%) (involved)	50.0 ± 10.5	53.1 ± 14.8	0.24

## Discussion

Our case series revealed an ACL graft retear rate in female basketball players of 9.4%. Some studies which reported results from patellar tendon and hamstring reconstruction also described incidence of hamstring ACL graft retear, but did not necessarily focus on graft ruptures [15,19-22]. According to these reports, the retear rate ranged from 2% to 8%. Salmon *et al*. concluded that risk factors of graft retear included return to competitive sports that require side-stepping, pivoting or jumping [15]. Their findings are consistent with our data with regard to the high incidence of retear in female basketball players.

Previous reports describe external and internal risk factors of primary ACL injury in female athletes [1,2]. External factors include the type of competition, shoe-surface interface, and muscle strength. Internal factors include anatomical, hormonal, and neuromuscular risk factors. However, risk factors of ACL graft retears remain unknown.

Salmon *et al*. reported incidence and risk factors of ACL graft rupture and contralateral ACL rupture over five years after reconstruction [15]. Repeated ACL injury occurred in 12% of the patients, and risk factors included a return to competitive sports that require side-stepping, pivoting, or jumping, as well as the contact mechanism of the index injury. Rate of graft ruptures and contralateral ACL ruptures were both 6%, although graft ruptures occurred significantly earlier than did contralateral ACL ruptures. According to their data, approximately 70% of all ACL graft ruptures occurred postoperatively, within a 24-month period [15]. Our data show that all graft retears were observed within the first 18 months. Furthermore, mechanisms of graft retear were completely different from those of the primary injury. While routine physical examination before re-injury revealed no instability in patients in the retear group, the remodeling phase of transplanted grafts is likely to continue throughout this period, and failures in graft maturation may influence retears. Based on these results, we would not recommend an early return to playing basketball.

Mean preoperative period of the retear group was shorter than that of the uninjured group, but the difference was not statistically significant. While we lack scientific evidence to support this, it is possible that a short preoperative duration might influence player condition. First, this could lower the likelihood that they will restore their physical condition including muscle strength, balance, and agility before ACL reconstruction. Second, it may influence their sense of fear. A long preoperative period seems to induce a sense of fear towards returning to their previous sports activity. However, in the absence of fear, patients do not hesitate to return to their sport after ACL reconstruction. As such, a shorter preoperative period may lead to ACL graft retear.

Mean age of the players was lower in the retear group than that in the uninjured group, but the difference was not statistically significant. However, all graft retears occurred in high school players. As young players are not supervised by an athletic trainer in most high schools, surgeons and physical therapists should provide stringent follow-up following ACL reconstruction.

Interestingly, preoperative quadriceps strength in the injured graft group was significantly lower than that in the uninjured group. A study by de Jong *et al*. revealed an association between preoperative quadriceps strength and postoperative functional performance [22]. The authors showed that an increased preoperative quadriceps deficit resulted in a lower postoperative function at six and nine months. In addition, they observed a quadriceps strength deficit of almost 20%, which persisted for one year. Residual quadricep weakness after ACL reconstruction has been shown in several studies [23-27]. Measured with a Cybex dynamometer, Keays *et al*. reported a 12% quadriceps strength deficit at 60° per second and a 10% deficit at 120° per second at six months [26]. Kobayashi *et al*. showed an approximate 10% quadriceps deficit postoperatively even after two years [25]. Furthermore, many reports on chronic ACL-deficiency cases found an association between postoperative quadriceps deficit and functional performance [23,27-29]. However, our case series found no significant difference in postoperative muscle strength between the uninjured and injured groups. Accurate clinical relevance of preoperative quadriceps deficit in ACL graft retears remains unclear at the present time.

We hypothesize that patients who showed strength deficits might have deficits in agility, balance, and proprioception. Rendstrom *et al*. noted that prevention of primary ACL injuries required a program which includes muscle strength and power exercises, neuromuscular training, and plyometrics and agility training [2]. When deficits in these elements were retained postoperatively, patients returned to basketball not fully healed and in unsafe conditions. Further studies are required to clarify if preoperative muscle weakness reflects deficits in other elements.

Limitations of this study include the lack of functional assessment, the relatively short duration of follow-up, and the small number of retear cases. We recognize the importance of functional assessment, as well as evaluation of muscle strength in patients prior to returning to sports activity. As data were incomplete in most patients, we excluded results of the functional tests from the present study. Regarding follow-up duration, further observation is required to demonstrate long-term results of ACL reconstruction. However, our data in the present study demonstrated that re-injury occurred within 18 months after the index surgery, representing the reality of ACL graft retears. Further cases are required to clarify the validity of the present study results.

## Conclusions

We examined risk factors of graft retear in female basketball players after anatomic double-bundle ACL reconstruction using hamstring autografts. ACL graft retears occurred in 9.4% of female basketball players in our study. Considering the timing and mechanism of graft retears, an early return to basketball should be avoided after ACL reconstruction. Preoperative conditions such as muscle strength and preoperative period, as well as postoperative status require close attention.

## Competing interests

The authors declare that they have no competing interests.

## Authors' contributions

YT drafted the manuscript. SH and YY contributed to study design and manuscript structure. TK, NS and ST contributed to muscle strength assessment. YS advised clinical opinions for assessing retear cases.

All authors have read and approved the final manuscript.
